# The neurobiological basis of ADHD

**DOI:** 10.1186/1824-7288-36-79

**Published:** 2010-12-22

**Authors:** Paolo Curatolo, Elisa D'Agati, Romina Moavero

**Affiliations:** 1 Department of Neuroscience, Pediatric Neurology Unit, "Tor Vergata" University of Rome, Italy

## Abstract

Attention-Deficit/Hyperactivity Disorder is not a single pathophysiological entity and appears to have a complex etiology. There are multiple genetic and environmental risk factors with small individual effect that act in concert to create a spectrum of neurobiological liability. Structural imaging studies show that brains of children with Attention-Deficit/Hyperactivity Disorder are significantly smaller than unaffected controls. The prefrontal cortex, basal ganglia and cerebellum are differentially affected and evidence indicating reduced connectivity in white matter tracts in key brain areas is emerging. Genetic, pharmacological, imaging, and animal models highlight the important role of dopamine dysregulation in the neurobiology of Attention-Deficit/Hyperactivity Disorder. To date, stimulants are the most effective psychopharmacological treatments available for Attention-Deficit/Hyperactivity Disorder. Currently only immediate release methylphenidate and atomoxetine are approved for the treatment of ADHD in Italy. Drug treatment should always be part of a comprehensive plan that includes psychosocial, behavioural and educational advice and interventions.

## Introduction

Attention-Deficit/Hyperactivity Disorder (ADHD) is a common, long-lasting, treatable childhood psychiatric disorder, characterised by a pattern of developmentally inappropriate inattention, motor restlessness, and impulsivity that affects approximately 3-7% of school-aged children [1].

ADHD was first recognised 100 years ago as a childhood disorder found mainly in boys, and was initially described as "hyperactivity" or "hyperkinetic disorder of childhood". This abnormal behaviour was found to be the result of a biological condition rather than a result of poor parenting [2]. In the 1960's and 70's much of the focus on what is now called ADHD was on hyperactivity. The presence of excessive movements in children was proposed to result from bilateral cortical activity secondary to a lack of transcallosal fibre tract-mediated interhemispheric inhibition [3]. Attention Deficit Disorder with or without Hyperactivity first featured in DSM-III in 1980 [4], and the more recent DSM-IV-TR provided updated ADHD criteria [5]. For a diagnosis of ADHD, symptoms need to occur often, have persisted for the past six months, and be maladaptive and incongruent with the individual's developmental level. Additionally, an ADHD diagnosis is only given if at least some of the behavioural symptoms were present before the age of 7 years, occur in more than one setting, and cause significant impairment in social and school functioning.

The renaming of the disorder, the subsequent focus on attention, and the clarification of three subtypes led to a range of neurocognitive and neurobiological hypotheses regarding the etiology and pathophysiology of ADHD within a more specific brain localisation. Furthermore, neurocognitive models of ADHD have become more refined, and one particular executive process, inhibition, is now considered to be a core deficit [6].

Current theories emphasise the central role of attentional and executive dysfunctions in children [7,8], as well as affective components involving emotional control and motivational processes [9]. A growing body of evidence supports a model in which multiple genetic and environmental factors interact during early development to create a neurobiological susceptibility to the disorder; the expression of which is mediated by alterations within different and diverse neural networks and deficits in the neuropsychological functions that these subserve [10]. Individuals with ADHD present difficulties in several domains of attentional and cognitive functions: problem solving, planning, orienting, alerting, cognitive flexibility, sustained attention, response inhibition, and working memory [7,11]. Other domains involving affective components, such as motivation and delay aversion, are also affected [8,9]. Motor difficulties, such as problems with sensory motor coordination, including poor handwriting, clumsiness, and marked delays in achieving motor milestones [12], have also been reported and the prevalence of motor impairment in the ADHD population has been estimated to be approximately 50% [13]. Motor problems might be partially related to abnormalities in structure and/or function of the cerebellum and basal ganglia found in ADHD [14].

Recently, neuroimaging has led to several important advances in the understanding of the neurobiology underlying the clinical picture of ADHD, and demonstrates a clear brain basis to the disorder in regions involved in attention, and executive and inhibitory control [15,16]. Furthermore, transcranial magnetic stimulation (TMS) has provided evidence that intracortical inhibition, as indexed by the immature ipsilateral motor cortex, normalises with psychostimulant treatment [17]. There is an exciting confluence between emerging studies in basic neurobiology and the genetic, neuroimaging, and neuropsychological analyses of ADHD. Knowledge of neurobiology can offer child neurologists, psychiatrists and other healthcare professionals a valuable framework for the interpretation of clinical findings of children meeting the criteria for diagnosis of ADHD. In this article we provide a brief overview of the salient neurological basis of the disorder.

### Etiology

ADHD is not a single pathophysiological entity and appears to have a complex etiology. Multiple genetic and environmental factors act together to create a spectrum of neurobiological liability.

### The genetic basis for ADHD

Genetic factors are implicated in ADHD, but the mechanism of action is not completely understood. Twin, family and adoption studies of ADHD have supported a strong genetic contribution to the disorder, with heritability ranging from 60-90% [18,19].

Genes regulating neurotransmitter systems have been implicated in ADHD. Candidate gene studies of ADHD have produced substantial evidence implicating several genes in the etiology of the disorder, with meta-analyses supportive of a role of the genes coding for DRD4, DRD5, SLC6A3, SNAP-25, and HTR1B [20]. Genome scan studies on potential alleles for ADHD have demonstrated linkage on chromosomes 5p13, 6q12, 16p13, 17p11 and 11q22-25 [21,22]. However, genome-wide association studies have failed to report any associations that are significant after correction for multiple testing [23]. Therefore, a plausible genetic hypothesis for ADHD is a mixture of dominant and recessive major genes that act with complex polygenic transmission patterns [18]. An increased rate of large, rare, chromosomal deletions and duplications known as copy number variants have been reported in individuals with ADHD [24]. However, genetic testing in an individual child is not currently practical in normal clinical practise.

Sometimes ADHD-like symptoms are exhibited by patients with established neurogenetic disorders such as Tuberous Sclerosis Complex, Neurofibromatosis I, Turner Syndrome, Williams Syndrome, Velocardiofacial syndrome, Prader-Willy syndrome, and Fragile × Syndrome. Although each syndrome may arise from different genetic abnormalities with multiple molecular functions, the effects of these abnormalities may give rise to common effects downstream in the biological pathways or neural circuits, resulting in the presentation of ADHD symptoms [25].

### The environmental basis of ADHD

Pre-, peri- and postnatal environmental factors play an important role in the pathogenesis of ADHD. Prenatal factors are associated with maternal lifestyle during pregnancy. For example, prenatal alcohol exposure is known to induce brain structural anomalies, especially in the cerebellum [26]. Children exposed prenatally to alcohol can become hyperactive, disruptive, impulsive, and are at an increased risk of a range of psychiatric disorders [27,28]. Maternal smoking produces a 2.7-fold increased risk for ADHD [29], and a dose-response relationship between maternal smoking during pregnancy and hyperactivity has been reported [30]. This may be due to an effect on nicotinic receptors, which modulate dopaminergic activity. Dopaminergic disruption is believed to be involved in the pathophysiology of ADHD [31,32].

Peri-natal factors have also been implicated, with a two-fold increase in ADHD in very low-birthweight children and an increased rate of pregnancy and birth complications in mothers of children later diagnosed with ADHD [33].

Among postnatal factors, a role for malnutrition and dietary deficiency in ADHD has been proposed. An imbalance of essential fatty acid (omega-3 and omega-6) intake has been suggested to be potentially involved in the development of ADHD [34], although further evidence is required to establish a role. Iron deficiency has been implicated in some cases [35]. Early deprivation of social environment during the postnatal period may also have significant effects.

### Gene-environment interactions

More complex models of the etiology of ADHD incorporating gene-environment interplay need to be considered. Recent studies have focused on the joint effects of gene variants (of DRD4 and DAT1) and prenatal substance exposures on subtypes of ADHD children, demonstrating that smoking during pregnancy is associated with the combined ADHD type in genetically susceptible children [36]. A significant interaction between DAT1 genotype and prenatal smoke exposure was found in males. Men homozygous for the DAT1 10-repeat allele had higher hyperactivity-impulsivity than males from all other groups [37]. Despite the heterogeneity of the etiology and pathophysiology of ADHD, abnormal DAT density seems to be common among subjects with ADHD [38].

### Neuroimaging

Growing evidence points to the involvement of the frontostriatal network as a likely contributor to the pathophysiology of ADHD. This network involves the lateral prefrontal cortex, the dorsal anterior cingulate cortex, and the caudate nucleus and putamen. In ADHD patients, reductions in volume have been observed in total cerebral volume, the prefrontal cortex, the basal ganglia (striatum), the dorsal anterior cingulate cortex, the corpus callosum and the cerebellum [39]. A developmental trajectories study in ADHD patients showed a delay in cortical maturation, and demonstrated that different clinical outcomes may be associated with different developmental trajectories in adolescence and beyond [40]. In studies of cortical development in children with ADHD, a marked delay in brain maturation was seen; the grey matter peaks were about 3 years later than in healthy controls [41]. The delay was most prominent in prefrontal regions important in the control of cognitive processes including attention and motor planning [41,42]. Compensatory networks including basal ganglia, insula and cerebellum have been implicated for relative lower cognitive load tasks in ADHD patients [43].

Neuroimaging studies have also reported reduced white matter (WM) volumes [43], midsagittal corpus callosum (CC) areas [44], and cortical thickness [43] in ADHD patients compared with controls. One of the most replicated alterations is a significantly smaller CC, but there are conflicting reports regarding the affected callosal segments [45]. Recent magnetic resonance imaging (MRI) structural investigations have shown that WM alterations are present in children, adolescents and adults with ADHD [46]. In 15 young males with ADHD, Silk *et al*. (2008) found WM abnormalities in several distinct regions underlying the inferior parietal, occipito-parietal, inferior frontal, and inferior temporal cortex [47]. Tractography methods showed that these regions form part of WM pathways connecting prefrontal and parieto-occipital areas with the striatum and the cerebellum. The authors also demonstrated anomalous WM development in ADHD in distinct cortical regions that they had previously shown to be dysfunctional or hypoactive in a functional MRI study of subjects with ADHD [47].

Diffusion tensor imaging (DTI) is an MRI modality that provides information about the direction and integrity of neural fibre tracks in the brain *in vivo*. DTI studies have revealed developmental changes in cortical WM pathways in prefrontal regions and in pathways surrounding the basal ganglia and cerebellum in patients with ADHD, which presumably reflect decreasing myelination of axons. It is believed that these changes cause a decrease in the speed of neuronal communication [48]. Moreover, the neural networks serving the corticostriatal and corticocerebellar circuits could represent putative biomarkers for ADHD. Indeed, in this disorder their quantification using DTI could be relevant for both diagnostic and therapeutic purposes [46].

As well as offering new data to map the brain systems involved in ADHD, and to integrate these findings with clinical symptoms, functional neuroimaging studies allow us to understand the mechanisms of treatment response [42,49]. Positron emission tomography (PET) studies have shown that methylphenidate hydrochloride (MPH) blocks dopamine active transporters (DAT) and that extracellular dopamine (DA) increases in proportion to the level of blockade and to the rate of DA release. This process is associated with an enhanced perception of the external stimulus as salient in subjects with ADHD [50].

### Clinical diagnosis and comorbidities

Clinical presentation of ADHD may vary according to age and stage of development and there are cultural differences in the level of activity and inattention that are regarded as a problem [51]. Diagnosis requires that there should be clear evidence of clinically significant impairment in social, academic, or occupational functioning [5]. The predominantly inattentive type is relatively more common in females. Children with the predominantly hyperactive-impulsive type are aggressive and impulsive, and tend to be highly rejected by their peers. The combined type causes more impairment in global functioning, in comparison with the other two types. Adolescents with ADHD often report low self-esteem and poor peer relationships; and are at high risk of smoking and substance abuse early in life [52,53].

Endophenotypes can be used as trait markers for disease susceptibility, to identify more genetically homogeneous subgroups, to highlight distinct pathophysiological mechanisms or etiological pathways, or to define "spectrum" phenotypes suitable for quantitative trait analyses [54]. Cognitive deficits and motor response inhibition are the prime endophenotype candidates in ADHD [55].

The co-existence of several other types of psychopathology along with ADHD, such as oppositional defiant disorder, mood and anxiety disorders, learning disorders, tics, and mental retardation, is very common [56].

### Treatment

Before starting treatment, it is important to identify the target outcomes to guide the therapy decision. Drug treatment should be based on a thorough assessment and should always be part of a comprehensive treatment plan that includes psychosocial, behavioural, and educational advice and interventions. Psychotherapy combined with medication may play a role in treating behavioural problems, organisational issues and psychiatric comorbidities [57]. In Italy, an ADHD diagnosis can only be made at a regional referral centre approved by the Italian Ministry of Health. Treatment guidelines put forward by the Ministry of Health and based on European guidelines, specify that pharmacological treatment can only be initiated after failure of cognitive behavioural therapy over a period of 6 months or longer has been demonstrated. Patients must first be enrolled in the ADHD medication registry before treatment with MPH or atomoxetine (ATX) can be prescribed.

Behavioural therapy and pharmacological treatment have both been shown to benefit ADHD patients. A longitudinal study of the efficacy of different treatments (an intensively monitored medication program, behavioural therapy, combination of medication and behavioural therapy or treatment as usual by community care) showed after 8-year follow-up that all four of the original treatment groups had a similar outcome: all showed improvement in comparison with pretreatment baseline scores, but none demonstrated superiority [58].

The fronto-subcortical circuits (lateral prefrontal cortex, dorsal anterior cingulate cortex, caudate, and putamen) associated with ADHD are rich in catecholamines, which are involved in the mechanism of action of medications used to treat this disorder. Neuropharmacological studies have provided evidence that ADHD involves dysregulation of both noradrenaline (NE) and DA neurotransmitter systems [59]. MPH treatment causes an increase in DA signalling through multiple actions, including blockade of the DA reuptake transporter, amplification of DA response duration, disinhibition of the dopamine D2 receptor and amplification of DA tone [60]. MPH is also an inhibitor of NE re-uptake. ATX is a selective inhibitor of synaptic re-uptake, and *in vivo*, it specifically increases extracellular levels of DA in the prefrontal cortex but not in the striatum; probably by modulating cortical synaptic DA uptake via the NE transporter [61]. Dextroamphetamine increases the synaptic activity of DA and NE by increasing the release of the neurotransmitters into the synaptic cleft, decreasing reuptake back into the presynaptic neuron, and inhibiting their catabolism [62]. Strong evidence exists indicating that stimulant medications, such as MPH and dextroamphetamine, and the non-stimulant ATX, are effective in improving ADHD symptoms [63]. Guanfacine is a selective alpha2A adrenergic receptor agonist, which improves working memory by stimulating postsynaptic alpha2A adrenoceptors, strengthening the functional connectivity of prefrontal cortex networks [64]. Guanfacine has also been shown to be effective in reducing ADHD symptoms [65,66]. Table 1 summarises the most important characteristics of these pharmacological treatments for ADHD. Only ATX and immediate release MPH are currently approved for the treatment of ADHD in Italy.

**Table 1:** Clinical characteristics of ADHD pharmacotherapies

Pharmacotherapy	**Molecular mechanisms **[59-66]	Formulations	Efficacy **(meta-analysis effect size) **[74,70]	Common adverse events [75-79]
***Stimulants***				

Methylphenidate	Blocks DA reuptake transporter, amplifies DA response duration, disinhibits D2 receptor, inhibits NE re-uptake	Immediate release	0.92 (0.80, 1.05)	Decreased appetite, insomnia, abdominal pain, headache dizziness, reduced weight gain, affective symptoms
		Osmotic release	0.90 (0.76, 1.05)	Decreased appetite, abdominal pain, headache
		Extended release	0.85 (0.65, 1.05)	Decreased appetite, headache, abdominal pain
		Long-acting	0.96 (0.75, 1.16)	Headache, insomnia, upper abdominal pain, decreased appetite, anorexia
		Transdermal patch	*Not available*	Appetite, nausea, vomiting, insomnia
		Dexmethylphenidate	0.76 (0.45, 1.08)	Decreased appetite, headache, abdominal pain, nausea

Dextroamphetamine	Increases release of DA and NE into synaptic cleft, decreases reuptake into presynaptic neuron, inhibits catabolism	Immediate release	1.24 (0.88, 1.60)	Decreased appetite, insomnia
		Extended release	1.13 (0.57, 1.69)	Palpitations, tremor, insomnia, decreased appetite, headache, dizziness, dry mouth, weight loss, abdominal symptoms
		Prodrug	1.52 (1.34, 1.71)	Decreased appetite, headache, insomnia, abdominal pain, irritability

Mixed amphetamine salts	Increases release of DA and NE into synaptic cleft, decreases reuptake into presynaptic neuron, inhibits catabolism	Immediate release	1.34 (0.95, 1.72)	Decreased appetite, agitation, insomnia
		Extended release	0.77 (0.59, 0.94)	Decreased appetite, headache, insomnia

***Non-stimulants***				

Atomoxetine	Selectively inhibits synaptic DA re-uptake	Immediate release	0.63 (0.57, 0.69)	Upper abdominal pain, decreased appetite, vomiting, somnolence, irritability, fatigue

Guanfacine	Selective alpha2A adrenergic receptor agonist	Immediate release	*Not available*	Sedation, insomnia, decreased appetite, dry mouth, constipation
		Extended release	0.8 (0.53, 1.07)	Somnolence, fatigue, upper abdominal pain, sedation

ADHD pharmacological therapies are generally well-tolerated (Table 1). However, concerns surrounding the cardiovascular safety of some of these drugs has prompted a recent examination of the effects of ATX and MPH on blood pressure (BP), heart rate (HR), and ECG parameters. MPH appears to cause minor increases in BP and HR, with no strong data to suggest that itincreases the QT interval. Limited data suggest that ATX may increase BP and HR in the short term; in the long term it appears to only increase BP. The effects of ATX on QT interval remain uncertain. Because the current evidence is based on research that has not been specifically designed to investigate the cardiovascular effects of these drugs, it is difficult to draw firm conclusions [67].

Both MPH and ATX significantly increase activation in key cortical and subcortical regions subserving attention and executive functions. Therefore, alterations in dopaminergic and noradrenergic function are apparently necessary for the clinical efficacy of pharmacological treatment of ADHD [68]. However MPH and ATX have both common and distinct neural effects, consistent with the observation that while many children respond well to both treatments, some respond preferentially to one or the other. Although pharmacotherapy for ADHD appears to prepare and facilitate the brain for learning, experiential programs need to elicit compensatory development in the brain. The clinical amelioration of some children after environmental experiential inputs and early cognitive/behavioural treatment could indicate outcome-associated plastic brain response [69]. One year of treatment with MPH may be beneficial to show enduring normalisation of neural correlates of attention. However, little is known about the long-term effects of stimulants on the functional organisation of the developing brain [70]. Recent findings have shown that chronic MPH use in drug-naive boys with ADHD enhanced neuropsychological functioning on "recognition memory" component tasks with modest executive demands [71]. Patients receiving pharmacological treatment for ADHD should always be closely monitored for both common and unusual potentially severe adverse effects.

## Conclusions

Convergent data from neuroimaging, neuropsychology, genetics and neurochemical studies consistently point to the involvement of the frontostriatal network as a likely contributor to the pathophysiology of ADHD. This network involves the lateral prefrontal cortex, the dorsal anterior cingulate cortex and the caudate nucleus and putamen [39]. Functional neuroimaging has provided new ways to examine the pathophysiology of ADHD, has shown widespread dysfunction in neural systems involving the prefrontal, striatal, and parietal brain regions, and has led to a brain model of deficits in multiple developmental pathways [72]. Molecular genetic studies support dysregulation of neurotransmitter systems as the basis of genetic susceptibility to the disorder, and it is becoming clear that the genotype may influence the response to medications [73]. Hopefully, advances in understanding the underlying neurobiology of ADHD will contribute to identifying more specific and targeted pharmacotherapies, and will help child neurologists to better manage their patients.

## Competing interests

Professor Paolo Curatolo has served as an Advisory Board Member for Eli Lilly and Shire, and has received research grants from Eli Lilly and Shire. The other authors have no conflicts of interest to declare.

## Authors' contributions

PC proposed and designed the study, and revised the final draft. EDA and RM reviewed all the relevant articles on the literature, and prepared the first draft under the supervision of Prof. Paolo Curatolo. All authors contributed to the intellectual content and approved the final version.
